# Unique Case of Epigastric Heteropagus Twins: A Surgical Challenge

**DOI:** 10.7759/cureus.82421

**Published:** 2025-04-17

**Authors:** Juhi Singhal, Sandeep Gupta, Varun K Agarwal, Akshay Brara, Neha Jain

**Affiliations:** 1 Surgery, Sarojini Naidu (SN) Medical College, Agra, IND; 2 Surgical Oncology, Sarojini Naidu (SN) Medical College, Agra, IND; 3 Pathology, Sarojini Naidu (SN) Medical College, Agra, IND

**Keywords:** conjoined twins, fetus-in-fetu, heteropagus twins, omphalocele, prenatal screening

## Abstract

Parasitic twins are an extremely rare condition, though their precise occurrence is unclear, with estimates suggesting a few reported cases. The parasite, the dependent, undeveloped twin, is attached to the independent, developed twin, called the autosite, at many sites. There are eight probable sites for attachment: the thoracopagus, omphalopagus, craniopagus, cephalopagus, parapagus, ischiopagus, pyopagus, and rachipagus. We report a very rare case of epigastric heteropagus in which the host’s epigastrium was attached to the parasite.

A 47-year-old woman presented with a progressive abdominal mass that had been present since birth. She had four alive children who were all delivered vaginally at home. The patient was found to be pale throughout general, physical, and systemic tests, with the remainder of her data being within normal ranges. Upon abdominal examination, a pedunculated enlargement measuring approximately 24 × 16 × 12 cm and exhibiting signs of a primitive face with a single ear, a nose, and limb buds was discovered. Contrast-enhanced computed tomography (CT) and CT angiography revealed a soft tissue swelling covering the patient’s anterior thorax and abdomen, measuring 6.7 × 13 × 7.5 cm in size. The patient’s right superior epigastric artery served as the primary vascular supply for the lesion.

A parasitic epigastric heteropagus twin is a rare congenital anomaly. In such cases, it is imperative to pre-operatively assess shared organs and the feeding arterial supply in order to excise the mass in toto without compromising the hemodynamics of the host.

## Introduction

Conjoined twins are delivered at a rate of one in 50,000-100,000 live births. Conjoined twins were referred to as heteropagus twins by Potter and Craig, who themselves were asymmetrical conjoined twins [1]. Parasitic twins are an extremely uncommon abnormality, the exact prevalence of which remains unknown but is estimated to be less than one in one million births [2-4]. The parasite, or the dependent, undeveloped twin, can attach to a number of different sites on the independent, developed twin, which is referred to as the autosite. The identifiable components of the parasite that are found in the fetus are typically attached to the automaton by a structure known as a pedicle, which is a soft tissue that contains major blood arteries [3,4]. Two main theories are currently considered to explain this phenomenon: the “fission” theory, which claims that the embryo is incompletely separated, and the “fusion” theory, which proposes that two sections that were once distinct come together to form a single entity [3-6]. The advanced theory postulates that a vascular compromise occurs in utero, which leads to either death or the resorption or partial resorption of one of the two twins [3-5]. There are eight possible attachment sites: the thoracopagus, omphalopagus, craniopagus, cephalopagus, parapagus, ischiopagus, pyopagus, and rachipagus. Fetus-in-fetu (FIF) is an abnormality that occurs when monochorionic, monozygotic, and diamniotic twinning occurs. This abnormality is the result of an uneven division of the totipotent inner cell mass of the growing blastocyst, which ultimately results in the inclusion of a smaller cell mass within a maturing sister embryo. In patients with FIF, a deformed parasitic twin is present, along with the presence of a spinal column and organs that range from rudimentary to well-formed and are arranged in a fetiform mass [1]. The incidence of FIF is one out of every 500,000 births. We report a case of epigastric heteropagus, a highly uncommon condition in which the epigastrium of the host is linked to the parasite.

## Case presentation

**Figure 1:**
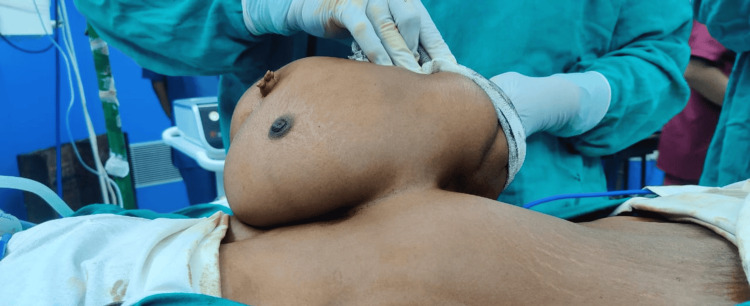
Pedunculated attachment of heteropagus twin

**Figure 2:**
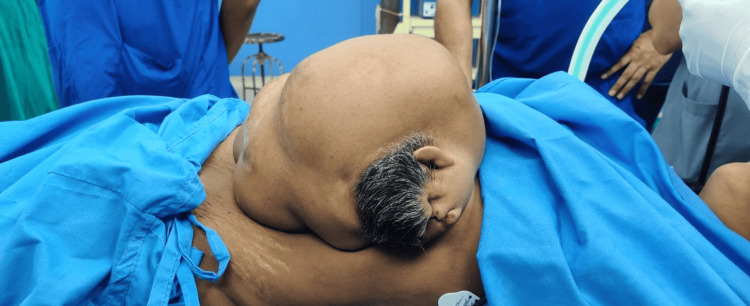
Heteropagus twin with visible scalp hairs and other facial features

**Figure 3:**
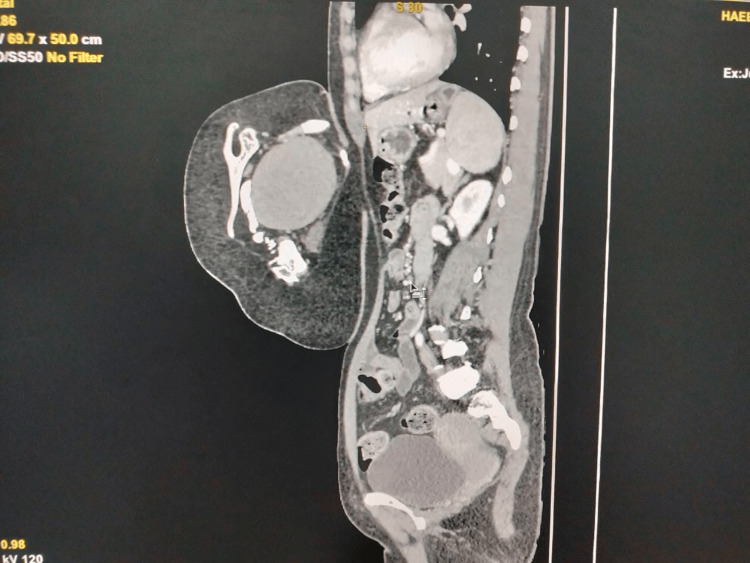
Skeletal structures such as the vertebral column and ribs are seen

A 47-year-old woman came in with an abdominal mass that had been present since birth and was progressively becoming bigger. It was associated with a dragging sensation, with no prior history of any urinary symptoms, postprandial distension, considerable weight loss, vomiting, nausea, or change in bowel habits. The patient has four alive children who were all delivered vaginally at home. The patient was found to be pale throughout general, physical, and systemic tests, with the remainder of her examination findings within normal limits. Upon abdominal examination, a pedunculated enlargement (Figure 1) measuring approximately 24 × 16 × 12 cm and exhibiting signs of a primitive face with a single ear, a nose, and limb buds was discovered (Figure 2). The swelling exhibited a varied consistency, a well-defined border, and an uneven surface. On evaluation, beta-human chorionic gonadotropin (β-hCG), alpha-fetoprotein, and routine blood investigations were within normal limits, with the exception of hemoglobin (Hb) (9 g/dL). Contrast-enhanced computed tomography (CT) and CT angiography revealed a soft tissue mass covering the patient’s anterior thorax and abdomen, measuring 6.7 × 13 × 7.5 cm in size. CT revealed clearly distinct viscera, including a liver, primitive single kidney, unilateral chest cage, scapula, and vertebrae (Figure 3), as well as fluid-attenuating structures along the lesion’s posterior face. There was a large opening in the patient’s anterior abdominal wall measuring approximately 8.9 × 5.5 cm, through which a pedicle-like structure could be observed passing between the patient and the lesion. Additionally protruding through the opening as a pedicle were the patient’s preperitoneal fat and the anterior portion of the liver. The patient’s right superior epigastric artery served as the primary vascular supply for the lesion. The patient underwent an excision of the mass in toto, with the removal of a part of the preperitoneal fat. The abdomen was closed in layers. The patient was discharged on postoperative day 12 after removing sutures. Histopathology examination confirmed the contents. The limb and vertebrae were identified on gross assessment, and histopathology revealed all components of the visceral organs and axial skeleton.

## Discussion

Monozygotic twins are a form of conjoined twins in which the inner cell mass does not split after the twins are united. In order to connect the embryos, a tissue bridge is used. Conjoined etiology can be explained by the incomplete division of the embryonic disc that occurs after the 13th day after the fusing of two monozygotic embryonic discs that were initially separate from one another. Some writers believe that the development of a parasitic twin is the result of selective ischemia damage that occurs in utero, which can either end in the death of the parasitic twin or its resorption, making it possible for the incomplete parasitic twin to become connected to the fully grown twin [7,8]. Both symmetrical and asymmetrical twins are possible, with the latter type of twins being referred to as heteropagus or parasitic twins throughout later stages of development. Further classification of these conditions can be made as follows: an externally attached parasitic twin, a contained FIF, and an interior teratoma. An acardiac connection is made by way of the placenta. Within the scope of our report, we discuss a case of epigastric heteropagus twins. Aberrant twining is the primary pathology of parasite twining, which is often believed to be monozygotic because of its prevalence. DNA testing has provided conclusive evidence for this assertion [9-11]. Based on our observations, we found a female patient who was 47 years old and had an automaton carrying a parasitic twin. The histological examination of her ovaries and fallopian tubes supported the diagnosis of monozygosity. Incorrect secondary neurulation in a closed neural tube represents another theory put forth as a possible cause of a heteropagus twin. According to this theory, the overproduction of the neural tube spills into the subcutaneous space and differentiates into distinct tissues, causing the development of rachipagus heteropagus, which is an uncommon condition that accounted for four out of the 39 cases in Sharma et al.’s review [3].

Based on our assessment of the literature, India has been found to have the highest linked occurrences of neural tube abnormalities [12-14]. The contents of the parasite revealed a primitive face that included hairs on the scalp, a single ear, a nose, and limb buds, all of which have been documented in previous studies [3,4,15]. Depending on the location where the parasite attaches itself, other challenges may occur. The detection of heart abnormalities in the autosite can be accomplished with the use of prenatal echocardiography [3,4]. Although there was no history of prenatal ultrasound in our case, it appears that the manner of birth is determined more by the anatomical configuration and location of the baby, rather than by the overall weight of the baby [4]. Additionally, it has been reported that a cesarean section was performed on heteropagus twins who each weighed 1,980 g due to dystocia [11]. In our case, the patient was delivered through a typical vaginal birth.

In most cases, the finding of a twin is made at delivery because of the apparent deformity that is visible to the naked eye. Excision through surgical means is performed as soon as the parasite is suspected of having an effect on the baby’s respiration and growth, in addition to contributing to the baby’s psychosocial burden and cosmesis. It has been noted that delayed presentations can occur at any age, including adolescence and even maturity [16]. In our case, the patient was reported to have been 47 years old, which is the oldest age ever documented at the time of surgery for a parasitic twin. It is possible that this case is the result of cultural concerns in developing countries, which often stigmatize children who have such deformities. There is no attempt made to provide treatment for the child in all cases, and they are kept concealed at home with little to no social interaction or attendance at school.

Ultrasound, CT, and magnetic resonance imaging can be used as pre-operative imaging modalities with the purpose of detecting such abnormalities [16]. Although the objective of imaging is to uncover connections between bone and soft tissues, it is important to note that when vascular connections are less complicated, angiography is not required. Although the vascular connections in adults may be complicated, it is feasible to exclude all potential arterial abnormalities in such cases with CT angiography, which may or may not be necessary. Echocardiography is recommended in all cases, as there is a higher prevalence of congenital cardiac disease in the autosite. When it comes to such cases, there is a significant amount of variety in anatomy, and the surgical procedure also varies. As no organ sharing is involved, the surgery is typically straightforward and uncomplicated [3,4,15]. The presence of bone and visceral unions can make excision challenging, as seen in our case, where the parasite and body tissue were linked to the xiphisternum of the autosite. In Sharma et al.’s review, bone and visceral unions were found in seven (18%) of the 39 cases [3]. As a result of the great size of the defect, the subsequent challenge is to close the defect after the parasite has been removed, which may necessitate the use of additional flaps or tissue expanders [3,4]. We were able to complete a primary closure in our case without having to go through these additional stages. The survival rate of autosite is very high, except for cases that have particularly severe cardiorespiratory issues [3,4]. The only cases in which autosite mortality occurred were 15 out of the 49 that were reported. On the 10th postoperative day, our patient was discharged with the possibility of morbidity development, such as wound infection, incisional hernia, and teratoma, all of which can be caused by such surgeries, which we have not yet encountered [3]. In a case report by Osama et al., a teenager was observed to have a mass in the retroperitoneal region, which was thought to be a teratoma; in contrast to what we observed in our case, the imaging carried out was unable to differentiate between the vertebrae and limbs. Histopathology was the only type of examination that verified the diagnosis of FIF [17].

## Conclusions

Heteropagus twin is a rare parasitic relationship between monochorionic, monoamniotic twins in utero. A failure of the complete development of the parasitic twin, while the same hindering the growth of the host, is a double-edged sword. Among the various types, epigastric heteropagus twins are most likely one of the rarest. In such cases, pre-operative imaging is necessary to rule out potential challenges intraoperatively and to determine whether the parasitic twin can be separated. It is imperative to have a proper pre-operative assessment of the organs shared and the feeding arterial supply in order to excise the mass in toto without compromising the hemodynamics of the host.
